# The effect of age-specific stay-at-home recommendation on healthcare utilization: Evidence from Finland’s COVID-19 policy

**DOI:** 10.1007/s10198-025-01887-z

**Published:** 2026-01-12

**Authors:** Jukka Laaksonen, Mika Kortelainen, Henri Salokangas

**Affiliations:** 1 INVEST Research Flagship Centre, Finnish Institute for Health and Welfare, Helsinki, Finland; 2 University of Turku, Turku, Finland

**Keywords:** Social distancing, Healthcare use, Quasi-experimental design, Regression discontinuity design, COVID-19, I10, I18, J14, D62, H12

## Abstract

Postponements of non-acute care during the COVID-19 pandemic commonly raised concerns about harmful health consequences and increased healthcare costs, particularly among older individuals. Using nationwide register data from Finland, we employ a regression discontinuity design to examine the effect of an age-specific stay-at-home recommendation on healthcare utilization during the first wave of the pandemic. We find that the recommendation reduced non-acute visits, such as dental care, physiotherapy, and specialized care visits, but had no effect on acute care use, including emergency department visits or inpatient stays. The reductions in dental care use were partly compensated for after the lockdown was lifted, but not in other non-acute services. Additionally, we find indicative evidence of a slight increase in mortality during the three-months post-period after the lockdown. Our findings suggest that a Scandinavian-type social distancing recommendation targeting the elderly may reduce non-acute healthcare use in the short term, thereby temporarily alleviating pressure on healthcare resources during a pandemic. However, the absence of rebound in some non-acute services highlight potential unmet needs, which may imply longer-term risks of functional decline, preventable hospitalizations, and associated healthcare costs. These findings point to the importance of policies that ensure continued access to essential non-acute care for older populations.

## Introduction

In early March 2020, World Health Organization (WHO) declared the coronavirus (COVID-19) outbreak a global pandemic due to its widespread distribution and severity [1]. Prior to the adoption of worldwide social distancing measures, several reports indicated that case-fatality rates among COVID-19 patients in China and Italy increased exponentially with age [2, 3]. Following the WHO’s emphasis on the heightened risk for older adults and individuals of all ages with pre-existing medical conditions [4], many governments implemented stay-at-home recommendations with varying levels of stringency, often specifically targeting older populations [5].

Prior studies have found reductions in healthcare utilization during the pandemic, especially among individuals without severe health issues [6–12]. These declines may be attributed to decreased mobility and the reduced spread of infectious diseases due to distancing policies, or to the intentional reduction of non-urgent activities aimed at preventing healthcare sectors from becoming overwhelmed. [13–18]. Postponements of non-acute care commonly raised concerns about preventable hospitalizations and increased healthcare costs, particularly among older adults at higher risk for ambulatory care sensitive conditions (ACSC) and functional decline [19–21]. These hospitalizations burden both patients and healthcare payers, including insurers and health authorities [19, 22, 23]. Thus, understanding how delays in healthcare access are related to later health outcomes is crucial for informing future healthcare policies that aim to ensure timely access to essential care and mitigate the risks of preventable hospitalization and worsening health conditions.

We study the effect of the Finnish age-specific stay-at-home recommendation on healthcare utilization, using extensive Finnish register data. In Finland, the government declared a state of emergency on March 16, during which individuals aged 70 and above were instructed to remain in quarantine-like conditions. The measure, commonly called as lockdown, was in effect until June 23, 2020. Using nationwide register data from Finland, we apply regression discontinuity (RD) design to compare individuals who were just over 70 years old when the policy was issued with those being just under 70 years old at the same time. Specifically, we examine the impacts on primary care, dental care, physiotherapy, non-acute specialized care and emergency department visits, as well as hospitalization. Building on earlier literature, we also explore effects on psychotropic drug and antibiotics use, and mortality during the lockdown. Additionally, we investigate potential heterogeneous effects for different subgroups, accounting for health risk factors for severe COVID-19 disease and region of residence.

Our study complements previous knowledge on the decline in healthcare service use in response to social distancing measures during the COVID-19 pandemic. Compared to earlier studies using quasi-experimental designs [13–15, 18, 24, 25], we utilize rich register data on the entire Finnish population and examine the effects of the age-specific recommendation on healthcare use at a remarkably detailed level. We aim to inform healthcare policies on the potential impact of delayed healthcare access on later health outcomes, such as functional disability, preventable hospitalizations, and mortality, particularly in the context of future pandemics or emergency scenarios.

In summary, we find that the Finnish age-specific stay-at-home recommendation reduced non-acute visits, including dental care, physiotherapy, and specialized care visits, but had no effect on acute care utilization, such as emergency department visits or inpatient stays. After lifting of the lockdown, we observe a partial rebound in dental care use, but not in other non-acute services. Additionally, we find indicative evidence of a slight increase in mortality during the three-months post-period after the lockdown. These findings suggest that a Scandinavian-type stay-at-home recommendation targeting older populations may reduce non-acute healthcare use in the short term, thus temporarily alleviating pressure on healthcare resources during a pandemic. However, the lack of rebound in some non-acute services raises concerns about unmet healthcare service needs, implying possible longer-term risks of functional decline, preventable hospitalizations, and related healthcare costs. These findings point to the importance of policies that ensure continued access to essential non-acute care for older populations.

## Background

### The first wave of COVID-19 in Finland

Followed by the deterioration of the coronavirus outbreak, the Finnish Government announced a state of emergency on 16 March 2020. Several non-pharmaceutical interventions (NPIs) were implemented including school, venue and public place closures, gathering restrictions and social distancing measures. Simultaneously, the potentially growing demand for healthcare and social welfare services was addressed by expanding service and coronavirus testing capacity, as well as decreasing non-urgent activities. In particular, preventative dental care was, in practice, completely discontinued [17]. As one of the distancing policies, individuals over 70 years were instructed to avoid contact with others, similar to quarantine conditions, except for parliament members, state leadership, and locally elected officials. This policy was justified by the fact that, compared to rest of the population, the elderly are at a greater risk of getting severe COVID-19 symptoms. In addition to elderly, individuals with underlying health conditions were also considered as part of the risk group. [26–28] As the Emergency Powers Act was enacted on 19 March 2020, restrictions on movement were tightened [29]. Particularly, the capital region (Uusimaa) was isolated on 28 March, and movement to and from the region was restricted due to high number of COVID-19 infections in the region, relative to rest of Finland (see Fig. 5). However, this restriction did not apply to mobility within Uusimaa, as the measures within the capital region were largely similar to those in the rest of Finland [30].

**Fig. 1:**
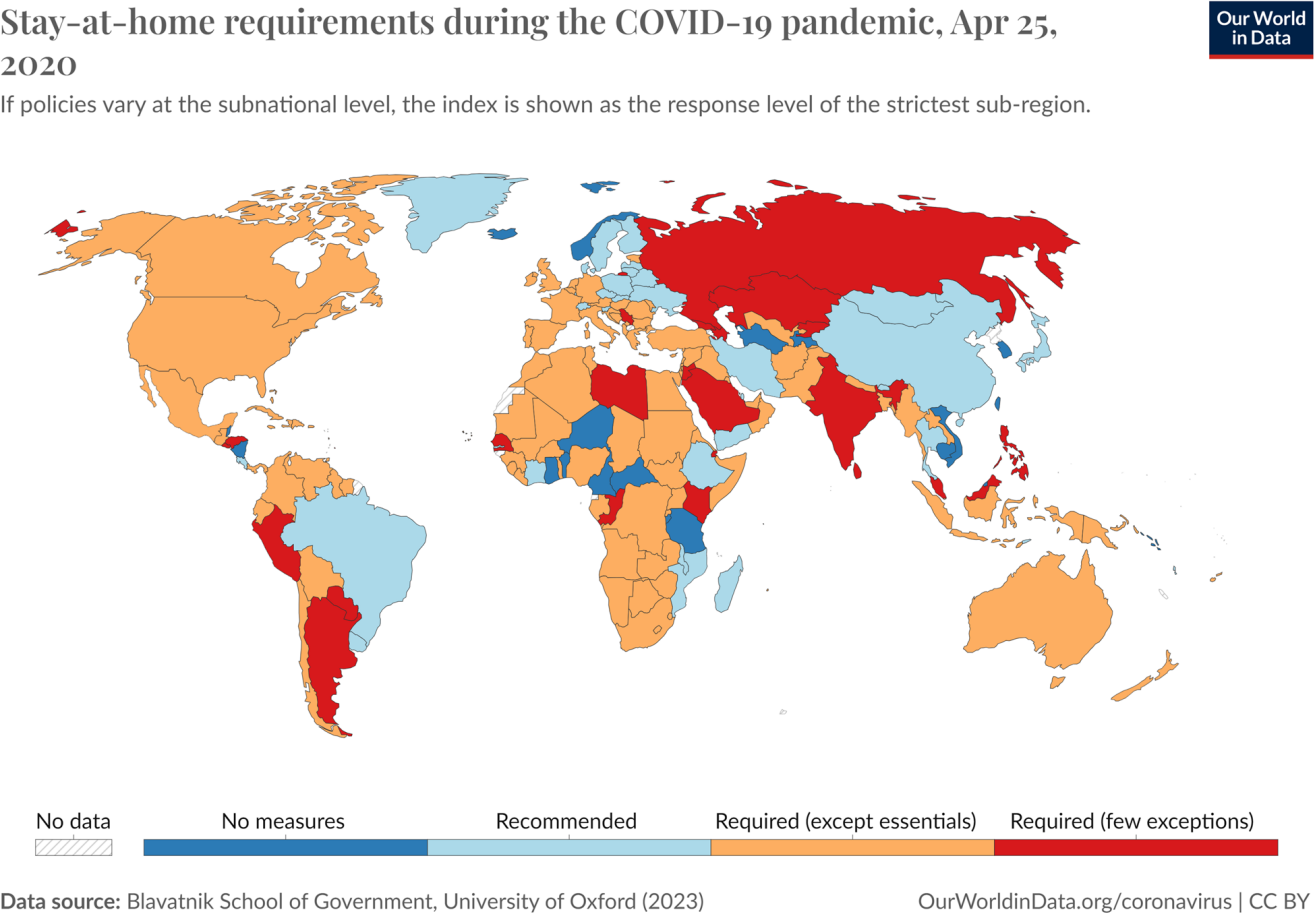
Strictness of stay-at-home requirements across countries in the middle of the first wave of COVID-19 pandemic [31]

Initially, the measures introduced on 16 March were in force until 13 April 2020. However, on 9 April the Finnish Government extended the policies to remain in force until May 13 [32]. As the exception, the movement restrictions concerning Uusimaa region were removed on April 14 [33]. In middle of May, other restrictions were gradually lifted. The social distancing recommendation for individuals over 70 years old and others in the risk group was rephrased to emphasize personal discretion on May 4 [34]. Also, The Finnish Institute for Health and Welfare (THL), the leading Finnish authority of health expertise and guidance, updated its guidance for elderly concerning protection against the virus on May 19. It stated, for instance, that reserved healthcare appointments or disease control visits should not be cancelled without a reason [35]. The distancing recommendation for elderly was eventually fully lifted on 23 June 2020 [36].

### The Finnish case in comparison to other countries

How does the Finnish case of stay-at-home recommendation compare with other countries? We are particularly interested in comparing the Finnish social distancing measures to those of Sweden and Turkey due to the similarities in the implemented measures, as well as in the research designs used in prior studies focusing on these two countries. [14, 15]. Figure 1 provides an overlook from the middle of the first wave on how strictness of stay-at-home requirements varied by country. We observe that Finland and Sweden resorted to recommendations, while Turkey and many other European countries took stricter measures to contain COVID-19 transmission [31].

**Fig. 2:**
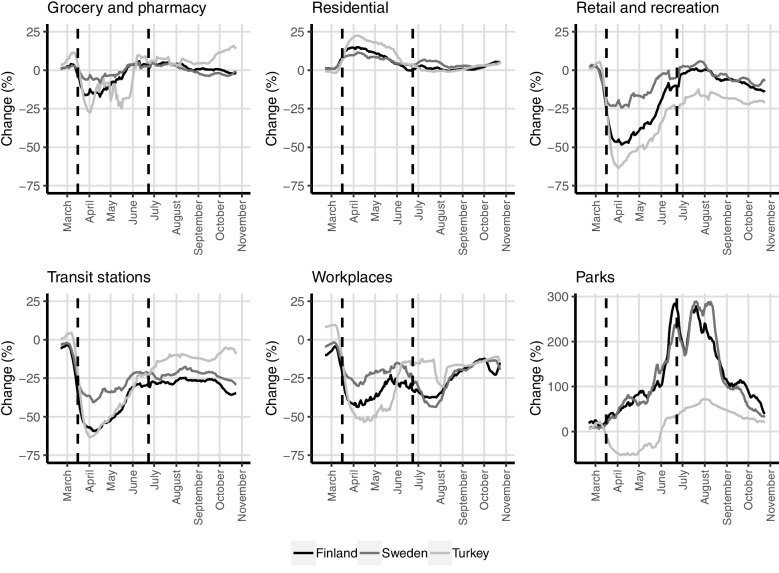
Changes in mobility during the first wave of COVID-19 in 2020, mobile phone location data by Google [37]. Notes: This figure displays the two-week rolling averages of relative changes in mobility for Finland, Sweden and Turkey, divided into six type of places: grocery and pharmacy, residential, retail and recreation, transit stations, workplaces, and parks. The vertical dashed lines mark the boundaries of the age-specific stay-at-home recommendation in Finland (March 16–June 23)

The mobility data based on mobile phone locations published by Google [37] provides additional insights. Figure 2 describes how mobility evolved in Finland, Sweden and Turkey during the lockdown period in Finland, 16 March to 23 June, 2020. Overall, in this comparison of three countries, the movement in public places appears to be declined the most in Turkey and least in Sweden. This may reflect the differences in COVID-19 policies between the countries. For instance, Turkish mobility restrictions imposed on elderly were mostly considered as a strict lockdown [15], while in Sweden and Finland stay-at-home measures were closer to recommendations [5]. In addition, the policies implemented in Sweden during the pandemic were relatively moderate compared to those in other Nordic countries as well [25], which may partially explain the mobility trends observed for Sweden. Based on this descriptive evidence, we assess that the general strictness of stay-at-home recommendations in Finland was somewhere between Sweden and Turkey.

## Methods

### Data

Our empirical analysis is based on Finnish nationwide register data on the entire Finnish population at the individual-level (N=5,525,037 at the end of 2019). We use three primary sources of data: FOLK-modules of Statistics Finland (including data on demographics, education and income); Care Register for Health Care (Hilmo) and Register of Primary Health Care Visits (AvoHilmo) of THL; and reimbursement registers of Social Insurance institution of Finland (KELA). Each dataset includes a unique individual identifier that Statistics Finland pseudonymizes before data access. These pseudonymized identifiers allow linkage of information across registers within Statistics Finland’s secure remote access environment, FIONA, ensuring both comprehensive data linkages and strict data protection. Our total study sample consists of all individuals aged 60–80 living in Finland at the time of the lockdown (N=1,292,452). We consider individuals born in March 1950 or before to be over 70 years old at the start of the lockdown. We exclude individuals who turned 70 during the lockdown (born in the second quarter of 1950) as their treatment status cannot be unambiguously determined (N=1,273,174 after the exclusion). Table 1 shows the descriptive statistics for all individuals aged 67–73, as this is the window within which we mainly produce our RD estimates.^1^

**Table 1:** Descriptive statistics for individuals aged 67–73

	Full sample	No health risk factors	Health risk factor
	(N=438,127)	(N=223,920)	(N=214,207)
*Baseline characteristics*			
Age	69.13 (1.80)	69.03 (1.80)	69.24 (1.80)
Female	0.52	0.57	0.48
Married	0.59	0.60	0.58
Lives alone	0.30	0.30	0.31
Resides in Uusimaa region	0.25	0.26	0.23
Completed upper secondary school	0.22	0.25	0.19
Foreign background	0.02	0.03	0.02
Disposable income (€)	22,470 (14,373)	23,083 (15,208)	21,830 (13,416)
Has a health risk factor	0.49	0.00	1.00
Ever used psychotropic drugs	0.48	0.42	0.55
*Pre-lockdown health care use*			
In-person primary care visit	0.36	0.29	0.44
Remote primary care visit	0.26	0.20	0.32
Public dental care visit	0.12	0.11	0.13
Dental care visit	0.24	0.24	0.24
Public dental care visit	0.12	0.11	0.13
Private dental care visit	0.12	0.13	0.11
Physiotherapy visit	0.03	0.03	0.04
Non-acute specialized care visit	0.15	0.08	0.22
Emergency department visit	0.04	0.03	0.06
Related to ACSC	0.006	0.003	0.010
Inpatient stay	0.04	0.02	0.06
Related to ACSC	0.007	0.002	0.012
Psychotropic drug use	0.14	0.09	0.19
Antibiotic use	0.08	0.06	0.10

Notes: This table displays baseline characteristics at the end of 2019 and pre-lockdown healthcare use (time period 1.12.2019–28.2.2020) for individuals aged 67–73. The descriptive statistics are presented within this particular window since the optimal windows selected in later analyses are less than three years for each outcome. Separate means are presented both for the full sample and sub-samples containing individuals with and without a risk factor for severe COVID-19 disease. Standard deviations for continuous variables are shown in parentheses. Ambulatory care sensitive conditions (ACSC) refer to such acute or chronic health conditions that have an increased risk of preventable hospitalization when appropriate treatment in primary care is not received [19, 22]. We use the classification of ACSC-related visits applied in a previous Finnish study [38], which also provides the detailed list of ICD-10 diagnosis codes used

Around half of the sample have a pre-existing medical condition that requires staying at home according to the recommendation, regardless of age. The risk group indicator is based on international COVID-19 related medical literature reviewed and summarized by THL [39]. We use this listing to identify the health risk group within our sample by exploring individuals’ diagnosis information and drug usage history. The baseline characteristics are additionally displayed at the month of birth level in Fig. 4, which shows that individuals just below and above age 70 appear to be very similar in terms of background characteristics.

We use multiple outcomes to study the impact of the age-specific recommendation (see full description in Appendix A.3). The pre-pandemic prevalence (3-month period prior to the lockdown) of the outcomes for ages 67–73 is described in the lower half of Table 1. To simplify interpretations, we code the outcomes as binary variables, indicating the probability of ever using a specific healthcare service or drug within a given time period.^2^

### Identification strategy

We apply RD design to identify the impact of the Finnish age-specific social distancing recommendation on healthcare usage. The Finnish social distancing recommendation targeted individuals aged 70 and above, making age a natural assignment mechanism. The outcomes of individuals just under 70 years old provide insights into how the outcomes of exposed individuals would have developed in the absence of the lockdown. This counterfactual is used to estimate the reduced form, representing the intent-to-treat (ITT) effect of the lockdown. Formally, the estimation is based on the following equation:

1 $$\begin{aligned} Y_i=\alpha +\tau D_i+f(X_i)+\epsilon _i, \end{aligned}$$

where $Y_i$ is the outcome for individual *i*, $D_i$ is the treatment indicator which gets the value of 1 for individuals who were at least 70 years old in March 2020 (i.e. born in March 1950 or before) and 0 otherwise. The running variable, which is age at the birth month level in our application, is represented by $X_i$ and $f(X_i)$ is a linear function of age which is fitted separately on both sides of the cutoff. The unobserved factors affecting the outcome $Y_i$ are captured by the error term $\epsilon _i$. We estimate the model using the local linear regression, applying kernel weights and using only observations within a chosen bandwidth. For binary outcomes, this corresponds to a local linear probability model, indicating that the coefficient *τ* can be interpreted as the change in probability (in percentage points) of the outcome at the cutoff. For each outcome, we apply the mean squared error (MSE) optimal bandwidth selection method and report conventional local-linear RD-estimates and robust bias-corrected confidence intervals using this bandwidth [40]. To ensure maximal precision of the estimates, we allow separate MSE-optimal bandwidths for each side of the threshold.^3^ We cluster the standard errors at the month-year of birth level.

The social distancing recommendation applied to two groups: individuals with underlying health conditions and those aged 70 and above. Assuming full compliance with these recommendations, all individuals in these risk groups would remain at home, while those outside these groups would determine their mobility at their own discretion. Under this assumption, the age-specific distancing recommendation would only impact individuals without underlying health conditions, as those with health risks would self-isolate regardless of age. Consequently, if this strict assumption holds, we would expect to observe discontinuities in healthcare utilization at the age-70 threshold only among individuals without underlying health conditions, while no such discontinuities should appear in the group with health risks.

However, this assumption may not hold in practice. First, descriptive reports from Finland suggest that there were no major differences in decreases in daily exercise or unmet healthcare service needs during the pandemic between those just below and above 70 years of age [41]. It is unlikely that the stay-at-home recommendation was followed to the letter; it rather directed individuals to avoid social contacts using personal discretion. Thus, instead of full compliance, we assume substantial non-compliance with the recommendation. Individuals with underlying health conditions aged 70 and above were subject to both the age-based and the health risk-based recommendations, whereas those under 70 with health risks faced only the risk-based recommendation. Consequently, if the compound of these two recommendations led to stronger compliance among individuals aged 70 and above, then discontinuities at the age 70 threshold could also appear within the health risk group.

Second, interpretation of age 70 threshold may vary across individuals. For instance, did people with no underlying health conditions, and who were about who turn 70 during 2020, identify themselves as being under the stay-at-home recommendation? An obvious interpretational problem arises in the group who turned 70 during the lockdown (born in the second quarter of 1950). While we exclude those who turn 70 during the lockdown and use this type of a donut-RD throughout the analyses, we acknowledge that the age-specific recommendation may also affect the social distancing behavior of even younger cohorts. Hence, we use uniform kernel in the estimation, which gives equal weight to each observation, and avoid overweighting these potentially ambiguous birth-month cohorts close to the threshold. However, we assess the sensitivity of our main results to bandwidth and kernel choice in Appendix A.6. To further consider the validity of the design, we present the results for three-months pre-lockdown period in Appendix A.4, showing that there are no clear differences in healthcare use between the treatment and control group prior to the lockdown.

Unfortunately, we are unable to address the concerns related to compliance since we do not observe any mobility outcomes at the individual level. Hence, we are not able to estimate the so-called first-stage effect of the social distancing policy on mobility. Observing the first-stage would provide us with information on how individuals in the target population have complied with the lockdown recommendation. Instead, our research design estimates the intention-to-treat (ITT) effect of the Finnish stay-at-home recommendation.

## Results

### Healthcare use during the lockdown

**Fig. 3:**
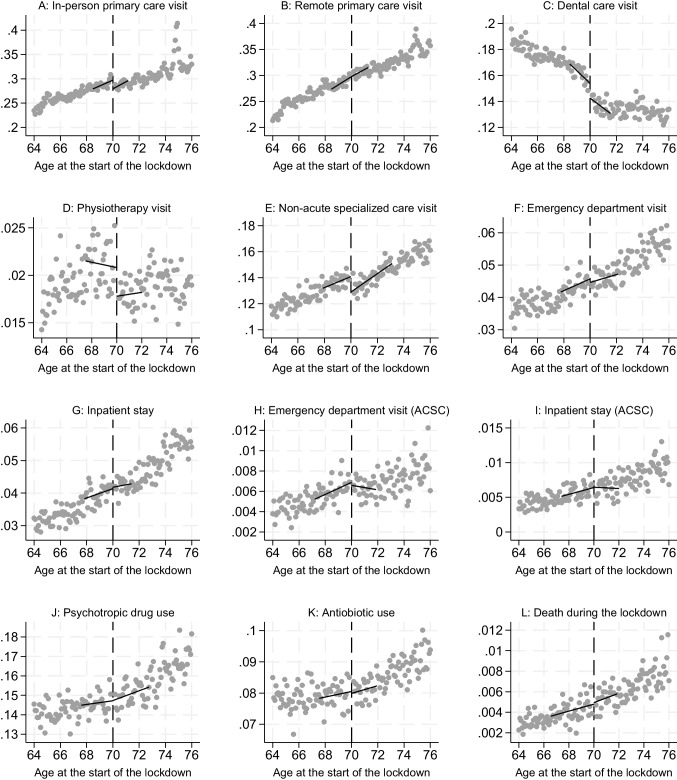
Impact of the lockdown on healthcare use and mortality. Notes: Each dot represents the average outcome of a particular birth month cohort. Individuals born in March 1950 or before are considered being over 70 years old at the start of the lockdown. Those born in the second quarter of 1950 have been excluded from the sample due to the ambiguous treatment status. The cutoff point at the age of 70 is described by the dashed line. The displayed linear fits are based on the MSE-optimal bandwidths used for the estimation, in which they are allowed to vary in length on each side of the threshold

**Fig. 4:**
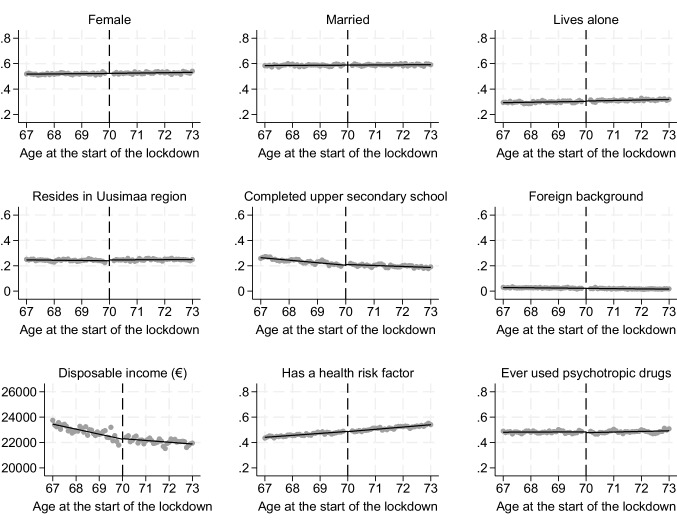
Balanced covariates. Notes: Each bin represents the average outcome of a particular birth month cohort. Individuals born in March 1950 or before are considered being over 70 years old at the start of the lockdown. Those born in the second quarter of 1950 have been excluded from the sample due to the ambiguous treatment status. The cutoff point at the age of 70 is described by the dashed line.

We begin by presenting the results through visualizations of age-based discontinuities in healthcare utilization at the age-70 threshold, as shown in Fig. 3. Each dot represents the average outcome during the lockdown period, based on age measured in months, excluding those turning 70 during the lockdown. For transparency, we plot each outcome over a broader age interval (64 to 76) to detect any potential heaps or strange patterns beyond the age-70 threshold. For interpretational clarity, the figures also include linear fits representing the estimated reduced form discontinuities.

Overall, we observe a linear relationship between age and healthcare utilization. The probability of healthcare use, drug use, and mortality increases with age, expect for dental care, which shows a negative correlation. At age 70 threshold, we find discontinuities in outcomes related to non-acute care (panels A to E), but not in acute care (panels F to I), drug use (panels J, K), or mortality (panel L). However, caution is warranted when interpreting the results for primary care visits (panels A, B). Based on visual exploration, we observe pronounced "heaps" in both type of primary care visits, particularly at age 75, but also at 65 and 70 in in-person visits. In Finland, routine health checkups and screenings are provided to older adults at certain ages, including, for instance, driving license checkup every five years. While we excluded some of these visits, we were unable to fully eliminate the systematic spikes at these intervals. Thus, the estimates for in-person and remote primary care visits are likely driven by these other factors unrelated to the stay-at-home recommendation. Supporting this interpretation, Table 8 shows that setting a placebo cutoff at age 75 produces a negative and statistically significant estimate for in-person primary care visits. However, we do not find such heaps, or statistically significant estimates at the placebo cutoffs, in other outcomes, suggesting that the observed discontinuities in non-primary care are plausibly explained by behavioral responses to the lockdown.

**Table 2:** Impact of the lockdown on healthcare use and mortality

	**A: In-person primary care visit**	**B: Remote primary care visit**	**C: Dental care visit**	**D: Physiotherapy visit**
Full sample	-.018** (-.033, -.004)	.000 (-.015, .014)	-.011** (-.017, -.001)	-.003** (-.006, .000)
Counterfactual outcome	.297	.298	.154	.021
Observations	170,561	177,219	200,304	298,594
No health risk factors	-.01 (-.032, .011)	-.001 (-.02, .015)	-.018*** (-.024, -.008)	-.002 (-.005, .001)
Counterfactual outcome	.238	.228	.159	.016
Observations	88,306	87,875	124,941	186,604
Health risk factor	-.026*** (-.043, -.016)	.008 (-.004, .025)	-.006 (-.016, .007)	-.007** (-.012, -.002)
Counterfactual outcome	.362	.371	.15	.028
Observations	69,393	97,783	95,329	116,498
	**E: Non-acute specialized care visit**	**F: Emergency department visit**	**G: Inpatient stay**	**H: Emergency department visit (ACSC)**
Full sample	-.012*** (-.019, -.005)	-.001 (-.005, .003)	.000 (-.003, .005)	.000 (-.001, .001)
Counterfactual outcome	.141	.046	.041	.007
Observations	359,445	293,788	231,892	314,397
No health risk factors	-.005 (-.012, .003)	-.001 (-.005, .003)	.000 (-.005, .005)	.000 (-.001, .001)
Counterfactual outcome	.086	.032	.024	.004
Observations	145,653	167,870	147,904	164,473
Health risk factor	-.014*** (-.026, -.006)	-.002 (-.007, .004)	.001 (-.004, .007)	-.001 (-.003, .001)
Counterfactual outcome	.198	.06	.06	.011
Observations	143,855	143,754	121,740	109,627
	**I: Inpatient stay (ACSC)**	**J: Psychotropic drug use**	**K: Antibiotic use**	**L: Death during the lockdown**
Full sample	.000 (-.002, .002)	.000 (-.008, .008)	-.001 (-.006, .005)	.000 (-.001, .001)
Counterfactual outcome	.006	.147	.08	.005
Observations	309,671	353,227	298,594	362,915
No health risk factors	.000 (-.002, .001)	-.001 (-.008, .006)	-.001 (-.007, .005)	.000 (-.002, .002)
Counterfactual outcome	.003	.102	.06	.002
Observations	169,069	193,510	192,579	181,509
Health risk factor	.000 (-.003, .004)	-.002 (-.011, .009)	.002 (-.005, .012)	.000 (-.002, .002)
Counterfactual outcome	.01	.197	.1	.008
Observations	127,589	190,573	129,768	194,420

Notes: ⁎p*<*0.1; ⁎⁎p*<*0.05; ⁎⁎⁎p*<*0.01. This table shows the conventional RD estimates within the MSE-optimal window, produced separately for each outcome using uniform kernel. The robust bias corrected confidence intervals are shown in parentheses, which are based on the standard errors clustered at the month-year of birth level. Note that the robust confidence intervals are not necessarily centered around the conventional RD estimates. Individuals born in the second quarter of 1950 have been excluded from the sample due to the ambiguous treatment status. The results are presented for the full sample and the sub-samples based on the health risk group status. For each estimate, the counterfactual outcome and the number of observations are displayed. The counterfactual outcome indicates the conventional local polynomial left estimate, which refers to the estimated outcome for the control group at the threshold. The results were derived with Stata using the rdrobust package [42]

Table 2 presents RD estimates for the full sample and the sample stratified by health risk status. The probability of in-person primary care visits declines statistically significantly by 1.8 percentage points, driven primarily by individuals in the health risk group. However, as noted above, this result is likely confounded and should be interpreted with caution. Additionally, we find significant declines of 1.1, 0.3 and 1.2 percentage points in the probabilities of dental care, physiotherapy and non-acute specialized care visits, respectively. For dental care, the decline is driven by individuals without a health risk factor, with Table 11 showing that the effect is particularly pronounced for the private dental care. In contrast, the reductions in physiotherapy and non-acute specialized care visits are primarily concentrated among those in the health risk group. It is also worth noting that when comparing the counterfactual outcomes in Tables 3 and 2, referring to the average outcomes for the control group at the threshold before and during the lockdown, we observe that the use of non-urgent care (A-E) is generally lower during the lockdown period, with the exception of remote primary care visits. This is in line with prior literature that has found reductions in in-person non-acute care use during the pandemic [7, 9–12] For the remaining outcomes – including acute care, mortality, and drug use – we find no statistically significant effects at the cutoff.

As discussed in Section“Identification strategy”, under the assumption of full compliance, the age-specific recommendation should have only affected individuals without health risk factors, as those with such conditions were advised to isolate regardless of age. However, we observe discontinuities not limited to the non-risk group but often more pronounced in the health risk group, suggesting incomplete compliance with the age-specific recommendation.

As part of our auxiliary analyses, we also examined post-lockdown effects. In Appendix A.5, we show that the decline in dental care visits was partially offset after the lockdown, with a 0.9 percentage point increase in visits during the three-month post-period among those exposed to the recommendation. Additionally, we find indicative evidence of a 0.1 percentage point increase in deaths during the post-period. This estimate is, however, partly sensitive to additional robustness checks, as discussed in Section“Robustness”. For other outcomes, we find no clear evidence of post-lockdown rebound effects.

**Fig. 5:**
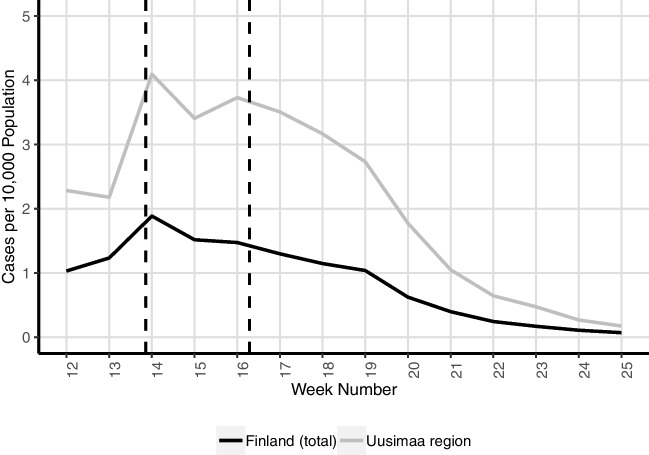
COVID-19 Cases per 10,000 Population in 2020. Notes: This figure displays the average number of COVID-19 Cases per 10,000 population in Finland and in the Uusimaa capital region over weeks 12–25 of 2020. The vertical dashed lines mark the boundaries of the isolation policy implemented for Uusimaa region (March 28–April 14, 2020)

Additionally, to account for regional variation, we conducted separate analyses for the Uusimaa region, which was subject to an isolation policy from 28 March to 14 April due to elevated infection rates during the early lockdown phase (see Fig. 5). During this period, movement to and from the capital region was restricted, while the measures applied within the region were broadly similar to those implemented elsewhere in Finland. (see Section“The first wave of COVID-19 in Finland”). We examine whether the age-specific recommendation affected healthcare use differently in the capital region–where the COVID-19 situation was more severe and the additional isolation measure was implemented–compared with the rest of Finland. According to Table 12, the observed reductions in dental care, physiotherapy, and non-acute specialized care appear to be driven by regions outside Uusimaa, where no significant effects are detected. In contrast, in Uusimaa, we observe declines of 1.0 and 0.2 percentage points in all emergency department visits and ACSC-related emergency visits, respectively. These findings suggest that the regional isolation measure and the overall more severe COVID-19 situation may have independently influenced acute care use, beyond the impact of the age-specific recommendation.

### Robustness

**Fig. 7:**
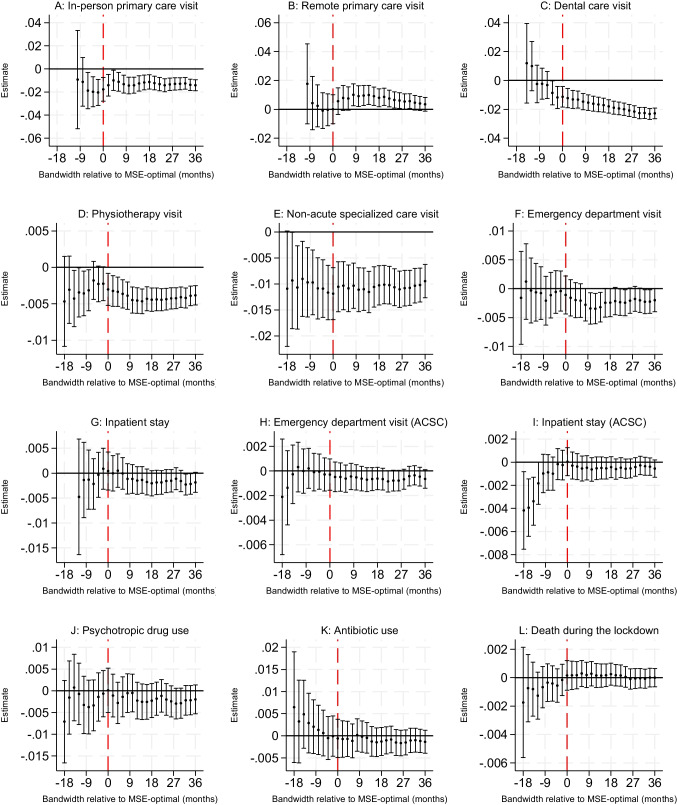
Robustness check: Main estimates during the lockdown period using alternative bandwidths relative to the MSE-optimal bandwidth. Notes: This figure shows conventional RD estimates and 95 percent confidence intervals for each outcome during the lockdown, using uniform kernel. The estimates are shown across several alternative bandwidths relative to the MSE-optimal. Those born in the second quarter of 1950 have been excluded from the sample due to the ambiguous treatment status

**Fig. 8:**
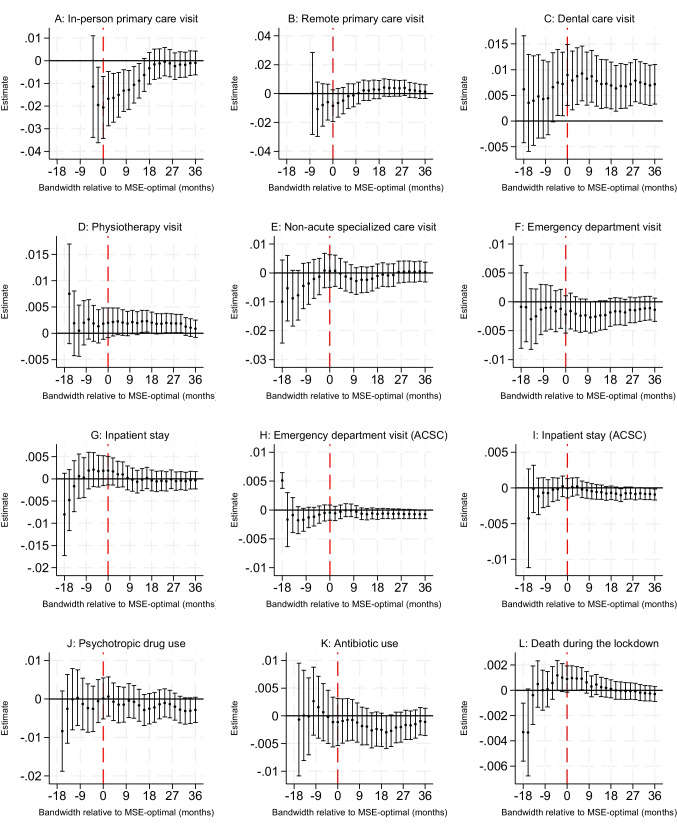
Robustness check: Main estimates during three-months post-period (Jul 2020–Sep 2020) using alternative bandwidths relative to the MSE-optimal bandwidth. Notes: This figure shows conventional RD estimates and 95 percent confidence intervals for each outcome during three-months post-period, using uniform kernel. The estimates are shown across several alternative bandwidths relative to the MSE-optimal. Those born in the second quarter of 1950 have been excluded from the sample due to the ambiguous treatment status

We perform several checks to ensure the robustness of our findings. First, Figs. 7 and 8 show that our main findings regarding dental care, physiotherapy, and non-acute specialized care visits remain relatively stable across multiple bandwidths in both lockdown and post-lockdown periods. However, the positive estimate for deaths during the post-period is somewhat sensitive to bandwidth choice, as the estimate shifts towards zero with wider bandwidths. Given also the relatively low overall prevalence of deaths, we consider the estimate for deaths during the post-period to be potentially uncertain.

Second, we report our main results using triangular kernel weights. The triangular kernel assigns weights to observations that decrease linearly with the distance from the cutoff, giving the greatest weight to observations closest to the threshold. According to Tables 6 and 7 the estimates for dental care visits and deaths during the post-period are partially sensitive to kernel choice, as they lose statistical significance when using a triangular kernel, despite showing effects of a similar magnitude. Placing greater weight on observations closest to the threshold may be problematic due to potentially varying interpretations of the recommendation among individuals close to the age 70. However, this highlights that when using highly granular monthly data, the transition in outcomes is relatively smooth exactly at the threshold– reflecting gradual behavioral adjustment – whereas analyses based on less granular data would likely display a more distinct discontinuity. While we consider the uniform kernel more suitable for our study setting, we acknowledge that interpreting the effect on dental care visits may be particularly challenging during the actual lockdown period, where we observed this type of smoothness.

Third, we perform placebo-treatment tests by conducting similar analyses with cutoffs at ages 65 and 75. Table 8 shows that apart from in-person primary care visits, which we consider biased due to routine health checkups and screenings (see discussion in Section“Healthcare use during the lockdown”), we do not observe any statistically significant differences at the level of five percent. These findings support the notion that our main results are plausibly driven by behavioral responses to the lockdown at age 70.

Fourth, as we rely on binary outcome variables, indicating whether a specific healthcare service was used at least once during a given period, we do not capture potential effects on service use intensity. To address this concern, we show the main results using count data in Table 9, reflecting the effect of the lockdown on the number of visits and drug purchases. These results are very similar compared to our main findings. In relative terms, the number of dental care, physiotherapy, and non-acute specialized care visits is decreased by 11%, 16%, and 12%, implying even larger effects than in our main results.

Finally, since we are examining several healthcare outcomes, we perform multiple hypothesis testing. In line with current practice in applied economics, we use the Romano-Wolf stepwise procedure to control the family-wise error rate (FWER) [43–45]. To maintain interpretability and limit the inflation of hypothesis tests, we organize outcomes into clinically coherent families: 1) Non-acute care, including in-person and remote primary care, dental care, physiotherapy, and non-acute specialized care visits; 2) Acute care, including emergency department visits (all-cause and ACSC) and inpatient stays (all-cause and ACSC); 3) Medication use, including psychotropic and antibiotic prescriptions; 4) Mortality, including deaths during the lockdown, treated as a distinct family and not pooled with utilization outcomes. Table 10 reports these results, confirming that our main estimates remain statistically significant even after accounting for multiple hypothesis testing.

## Conclusions

This paper provides new evidence on the impacts of age-specific social distancing measures during the COVID-19 pandemic. We examine how the Finnish government’s stay-at-home recommendation targeting individuals aged 70 and above affected healthcare utilization during the first wave of the pandemic. This research is particularly relevant in understanding how delaying healthcare access may affect older populations who are at higher risk of preventable hospitalization and other severe health outcomes.

We use a regression discontinuity (RD) design to investigate the impact of the lockdown on healthcare utilization. The results show a reduction of 1.1 percentage points in dental care visits (-7.1 %), 0.3 percentage points in physiotherapy visits (-14.3 %), and 1.2 percentage points in non-acute specialized care visits (-8.5 %) visits during the lockdown among individuals aged 70 and older. No significant effects are observed on emergency department visits, hospitalizations, mortality, or drug use. However, we find that the use of dental care was partially rebounded after lifting of the lockdown, as it was 0.9 percentage points (+4.1 %) higher during the three-months post-period among those who had been exposed to the stay-at-home recommendation. Additionally, we find suggestive evidence of a slight increase in mortality after lifting of the lockdown.

We do not find clear differences between individuals with and without health risk factors. Dental care visits declined more among those without risk factors, while reductions in physiotherapy and non-acute specialized care were driven by the risk group –suggesting non-compliance with the recommendation. In Uusimaa, where an additional isolation measure was implemented, we find no discontinuities in non-acute care at the threshold, but some declines in acute care use. These region-specific patterns may reflect the effects of isolation policies or differing responses from health authorities and residents, complicating interpretation.

Overall, we find minor impacts of the age-specific stay-at-home recommendation on non-acute care use, but no effects on acute care or drug use. The overall strictness of stay-at-home recommendations in Finland was somewhere between Sweden and Turkey, though closer to Sweden. While our findings largely align with the Swedish evidence, which found no effects in healthcare visits at the age 70 threshold [14], we find evidence of minor declines in certain areas of the healthcare sector in Finland. However, the results regarding psychotropic drug use contrast with survey-based findings from Turkey, where stricter lockdown for the elderly led to pronounced decreases in mental health outcomes [15]. While our outcome of drug purchases may also partially reflect supply, not only demand, the contrasting findings are likely related to differences of the stay-at-home policies between Finland and Turkey. More broadly, our findings are consistent with prior descriptive evidence showing a decline in non-urgent healthcare use during the pandemic and the partial rebound following the first wave. [6, 7, 9–12] On the other hand, the relatively low number of COVID-19 cases and deaths in Finland during the first wave, compared with the EU average [46], may partly explain the lack of reducing effect of the lockdown policy on acute care use and mortality, which has been reported in some previous papers [14, 21, 25].

Our contribution lies in a detailed analysis of healthcare service use during the first wave of COVID-19 pandemic among elderly in Finland. To our knowledge, no previous study has reported an age-specific decline in healthcare use due to social distancing with this level of detail, although the healthcare use of older adults during the pandemic has been widely studied [7, 14, 18, 47–50]. More generally, this examination contributes to the existing literature on the association between delayed healthcare access and subsequent health outcomes [51–53]. This information is essential for healthcare policies aiming to ensure access to necessary care and reduce the risk of avoidable hospitalizations, functional decline, and other adverse health outcomes, particularly among high-risk groups.

Perhaps the most important limitation of our paper is the lack of individual-level mobility data, making the estimation of the compliance with the stay-at-home recommendation challenging. Additionally, our reliance on register data may overlook more qualitative aspects, such as mental health impacts beyond psychotropic drug use, or delayed health consequences that could manifest after the follow-up observed. Finally, while the RD design is theoretically robust for age-based policy evaluation, the likely high rate of non-compliance complicates the interpretations.

Our findings suggest that a Scandinavian-type stay-at-home recommendation aimed at older populations can temporarily reduce the use of non-acute healthcare, thereby easing short-term pressure on healthcare resources during a pandemic. At the same time, the absence of a rebound in some non-acute services indicates potential unmet healthcare service needs that might translate into longer-term risks of functional decline, preventable hospitalizations, and additional healthcare costs. These findings point to the importance of maintaining access to essential non-acute care for older populations, even amid mobility restrictions.

## Figures and Tables

**Fig. 6:**
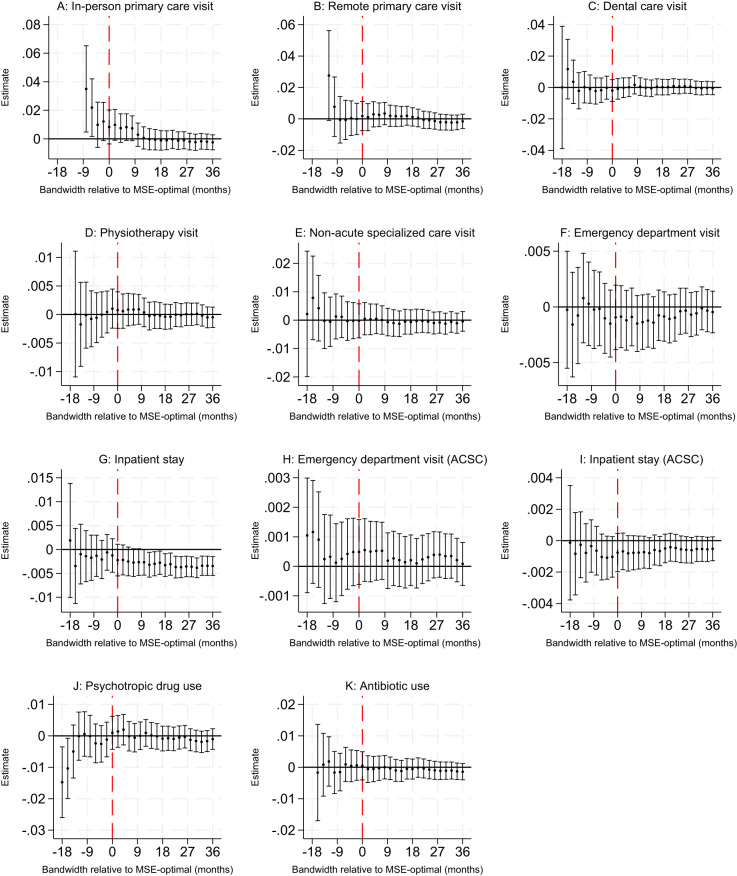
Robustness check: Main estimates during three-months pre-period (Dec 2019–Feb 2020) using alternative bandwidths relative to the MSE-optimal bandwidth. Notes: This figure shows conventional RD estimates and 95 percent confidence intervals for each outcome during three-months pre-period, using uniform kernel. The estimates are shown across several alternative bandwidths relative to the MSE-optimal. Those born in the second quarter of 1950 have been excluded from the sample due to the ambiguous treatment status

## Data Availability

The analysis utilizes licensed data from Statistics Finland and FinData. Access to the data is not limited to a specific institution or research group. Researchers from universities and research institutes within the EU can use the data through Statistics Finland’s remote access system, provided they meet the required data security approvals. Information on the application process can be found on the Statistics Finland and Findata websites.

## Appendices

### A.3 Outcomes

We study several outcomes to address different aspects of healthcare use. First, the distinction between in-person visits (provided by a physician or other healthcare professional at a healthcare facility) and remote visits (service given remotely through telephone or video communication) is particularly relevant as prior reports suggest that demand for in-person primary care services decreased, while the use of remote services increased at the start of the first wave [54]. The data on primary care services is derived from Register of Primary Health Care Visits (AvoHilmo), which includes only visits in public healthcare centers. Private primary care services are obtained from the register for physician and dentist fees by procedure from KELA. In our main analyses, we combine public and private visits, measuring whether an individual has used any primary care services.

Second, prior studies have shown that, in Finland, the use of private dental care services is more common among elderly, as well as those with higher income and education [55, 56]. Besides visits on public dental care (AvoHilmo), we utilize the data on reimbursements of private dental care visits (Physician and dentist fees by procedure from KELA). In our main analyses, we examine dental care visits as a combined measure of public and private visits, but also present the separate estimates in the appendices.

Third, previous literature has highlighted physical disabilities and unmet needs for physical therapy as significant risk factors for hospitalization, particularly among older individuals at greater risk of functional decline [20, 57, 58]. To address concerns related to access to rehabilitation services and potential gaps in physical care for older individuals during the pandemic, we examine physiotherapy visits. We obtain these visits from Register of Primary Health Care Visits, which contains physiotherapy visits to public providers.

Fourth, we investigate specialized care visits (Hilmo) by dividing the examination into non-acute visits, emergency department visits, and inpatient stays. Additionally, we separately study certain emergency department visits and inpatient stays that could have been potentially prevented by early access to a healthcare services. Ambulatory care sensitive conditions (ACSC) refer to such acute or chronic health conditions that have an increased risk of preventable hospitalization when appropriate treatment in primary care is not received [19, 22]. We identify such visits and inpatient stays based on the main diagnosis related to these events. The identified diagnoses are related to vaccine preventable conditions, such as bacterial pneumonia, influenza and immunization-related conditions; chronic conditions, such as hypertension and diabetes; and acute conditions, such as severe infections and dental conditions [38]. Moreover, motivated by previous literature [14, 25, 59, 60], we also study mortality during the lockdown.

Finally, through psychotropic drug use we aim to address potential mental health related issues related to increased isolation previously highlighted in literature [15, 61, 62]. Additionally, divergent results on antibiotic use during the pandemic [63, 64] motivates us to examine whether differences in antibiotic use can be observed close to the threshold.

### A.4 Pre-lockdown differences in healthcare use

To validate the study setting, we perform our analysis for three-months pre-lockdown period. That is, we use the same study population as in our main analyses and set the age-cutoff similarly, but instead consider outcomes for period from December 2019 to February 2020. Intuitively, we should not observe any remarkable discontinuities during the pre-period, as the age-specific social distancing recommendation had not yet been given. Estimates for the pre-lockdown differences are presented in Table 3. We do not find evidence on remarkable differences between the treatment and control group in healthcare or drug use during the pre-lockdown period. These findings are not sensitive to bandwidth (Fig. 6) or kernel (Table 5) choice.

**Table 3:** Differences in health care use and mortality during the three-months pre-period (Dec 2019–Feb 2020)

	**A: In-person primary care visit**	**B: Remote primary care visit**	**C: Dental visit**	**D: Physiotherapy visit**
Full sample	.008 (-.003, .027)	.002 (-.008, .011)	-.002 (-.011, .007)	.001 (-.002, .003)
Counterfactual outcome	.382	.267	.235	.032
Observations	148,389	189,044	296,175	241,911
No health risk factors	.011 (-.008, .033)	.006 (-.009, .02)	-.006 (-.018, .005)	-.001 (-.005, .003)
Counterfactual outcome	.307	.203	.242	.026
Observations	102,523	102,523	163,843	181,982
Health risk factor	-.001 (-.009, .015)	-.004 (-.02, .013)	.003 (-.007, .015)	.003* (-.001, .008)
Counterfactual outcome	.461	.335	.228	.039
Observations	76,094	89,618	148,821	111,411
	**E: Non-acute specialized care visit**	**F: Emergency department visit**	**G: Inpatient stay**	**H: Emergency department visit (ACSC)**
Full sample	.000 (-.008, .007)	-.001 (-.004, .002)	-.002 (-.005, .002)	.000 (-.001, .002)
Counterfactual outcome	.149	.042	.043	.006
Observations	267,782	346,201	279,932	364,050
No health risk factors	.005 (-.003, .014)	.001 (-.003, .004)	-.001 (-.004, .003)	.000 (-.001, .001)
Counterfactual outcome	.08	.026	.022	.003
Observations	133,497	133,199	169,251	163,455
Health risk factor	-.005 (-.021, .008)	-.003 (-.009, .003)	-.002 (-.008, .004)	.001 (-.001, .003)
Counterfactual outcome	.221	.059	.064	.009
Observations	117,771	152,255	134,906	162,164
	**I: Inpatient stay (ACSC)**	**J: Psychotropic drug use**	**K: Antibiotic use**	
Full sample	-.001 (-.003, .000)	.001 (-.006, .008)	.000 (-.004, .005)	
Counterfactual outcome	.007	.138	.078	
Observations	339,934	317,757	284,673	
No health risk factors	.000 (-.001, .000)	.000 (-.007, .006)	.000 (-.005, .005)	
Counterfactual outcome	.002	.093	.058	
Observations	135,886	172,209	163,387	
Health risk factor	-.002 (-.005, .001)	.001 (-.008, .013)	.000 (-.007, .009)	
Counterfactual outcome	.012	.185	.101	
Observations	138,685	182,183	138,301	

Notes: ⁎p*<*0.1; ⁎⁎p*<*0.05; ⁎⁎⁎p*<*0.01. This table shows the conventional RD estimates for the three-months pre-period within the MSE-optimal window, produced separately for each outcome using uniform kernel. The robust bias corrected confidence intervals are shown in parentheses, which are based on the standard errors clustered at the month-year of birth level. Note that the robust confidence intervals are not necessarily centered around the conventional RD estimates. Individuals born in the second quarter of 1950 have been excluded from the sample due to the ambiguous treatment status. The results are presented for the full sample and the sub-samples based on the health risk group status. For each estimate, the counterfactual outcome and the number of observations are displayed. The counterfactual outcome indicates the conventional local polynomial left estimate, which refers to the estimated outcome for the control group at the threshold. The results were derived with Stata using the rdrobust package [42].

### A.5 Healthcare use after lifting of the lockdown

The stay-at-home recommendation directed at individuals over 70 years old was fully lifted on 23 June 2020. To assess the potential rebounding effect of lifting the lockdown on healthcare use, we additionally conduct similar analyses for the period following the lockdown. For consistency, we limit these examinations to a time period of similar length as the lockdown. Additionally, extending the time window would introduce interpretational challenges due to potential bias from the second wave of the pandemic. Thus, we study a three months post-period from July to September 2020.

In our main analyses, we observed a discontinuity at the age of 70 in dental care, physiotherapy, and non-acute specialized care visits during the lockdown. Hence, we are particularly interested in whether this potential postponement of non-acute services is reflected in higher service utilization after the lockdown, compared with those who were not exposed to the stay-at-home recommendation. According to Table 4, the probability of dental care visits is increased by 0.9 percentage points during the post-period. We do not observe similar effects for physiotherapy or non-acute specialized care visits. Instead, we found some indicative evidence of 0.1 percentage points increase in deaths during the post-period. However, this estimate is partially sensitive to bandwidth and kernel choice, as shown in Appendix A.6.

**Table 4:** Differences in health care use during the three-months post-period (Jul 2020–Sep 2020)

	**A: In-person primary care visit**	**B: Remote primary care visit**	**C: Dental care visit**	**D: Physiotherapy visit**
Full sample	-.021** (-.047, .000)	-.008*** (-.019, -.003)	.009*** (.003, .017)	.002 (-.001, .005)
Counterfactual outcome	.345	.265	.217	.027
Observations	116,481	141,865	355,929	272,542
No health risk factors	-.02** (-.044, -.001)	-.002 (-.016, .007)	.004 (-.008, .014)	.001 (-.003, .005)
Counterfactual outcome	.287	.207	.223	.019
Observations	69,770	88,107	121,078	141,632
Health risk factor	-.015* (-.043, .003)	-.008* (-.02, .001)	.005 (-.008, .017)	.001 (-.005, .006)
Counterfactual outcome	.404	.326	.215	.036
Observations	71,565	82,060	137,581	121,878
	**E: Non-acute specialized care visit**	**F: Emergency department visit**	**G: Inpatient stay**	**H: Emergency department visit (ACSC)**
Full sample	.001 (-.008, .009)	-.002 (-.007, .002)	.002 (-.001, .005)	.000 (-.002, .001)
Counterfactual outcome	.149	.048	.039	.006
Observations	298,266	320,899	290,808	283,664
No health risk factors	.002 (-.005, .009)	.002 (-.004, .007)	.002 (-.001, .004)	.000 (-.001, .002)
Counterfactual outcome	.093	.033	.025	.003
Observations	180,698	215,998	219,599	179,285
Health risk factor	-.003 (-.02, .011)	-.006** (-.012, 0)	-.002 (-.007, .003)	-.001 (-.003, .002)
Counterfactual outcome	.211	.064	.058	.010
Observations	137,581	136,622	169,147	139,851
	**I: Inpatient stay (ACSC)**	**J: Psychotropic drug use**	**K: Antibiotic use**	**L: Death during the post-period**
Full sample	.000 (-.001, .002)	.000 (-.007, .008)	-.001 (-.006, .004)	.001** (.000, .002)
Counterfactual outcome	.006	.137	.067	.003
Observations	247,557	313,934	259,959	240,733
No health risk factors	.001 (0, .002)	.002 (-.004, .008)	.002 (-.003, .009)	.001 (-.001, .002)
Counterfactual outcome	.002	.093	.048	.001
Observations	147,449	186,204	137,354	182,723
Health risk factor	-.001 (-.003, .002)	-.003 (-.012, .009)	-.003 (-.011, .005)	.001 (.000, .003)
Counterfactual outcome	.010	.183	.086	.005
Observations	160,922	200,895	125,668	144,902

Notes: ⁎p*<*0.1; ⁎⁎p*<*0.05; ⁎⁎⁎p*<*0.01. This table shows the conventional RD estimates for the three-months post-period within the MSE-optimal window, produced separately for each outcome using uniform kernel. The robust bias corrected confidence intervals are shown in parentheses, which are based on the standard errors clustered at the month-year of birth level. Note that the robust confidence intervals are not necessarily centered around the conventional RD estimates. Individuals born in the second quarter of 1950 have been excluded from the sample due to the ambiguous treatment status. The results are presented for the full sample and the sub-samples based on health the risk group status. For each estimate, the counterfactual outcome and the number of observations are displayed. The counterfactual outcome indicates the conventional local polynomial left estimate, which refers to the estimated outcome for the control group at the threshold. The results were derived with Stata using the rdrobust package [42]

### A.6 Robustness

**Table 5:** Robustness check: Differences in health care use and mortality during the three-months pre-period (Dec 2019–Feb 2020), using triangular kernel

	**A: In-person primary care visit**	**B: Remote primary care visit**	**C: Dental care visit**	**D: Physiotherapy visit**
Full sample	.007 (.005, .025)	-.001 (.011, .009)	-.001 (.01, .008)	.001 (.001, .004)
Counterfactual outcome	.382	.27	.235	.032
Observations	212,198	208,145	371,621	315,498
No health risk factors	.012 (.006, .035)	.007 (.007, .023)	-.007 (.021, .004)	-.003* (.009, 0)
Counterfactual outcome	.309	.203	.242	.025
Observations	126,747	129,801	184,618	145,834
Health risk factor	.002 (.006, .014)	-.007 (.025, .01)	.004 (.006, .015)	.003* (0, .008)
Counterfactual outcome	.458	.338	.228	.04
Observations	105,828	116,498	185,694	140,042
	**E: Non-acute specialized care visit**	**F: Emergency department visit**	**G: Inpatient stay**	**H: Emergency department visit (ACSC)**
Full sample	.000 (.007, .008)	-.001 (.004, .002)	-.002 (.006, .003)	.001 (.000, .002)
Counterfactual outcome	.149	.042	.043	.006
Observations	340,693	441,136	353,549	456,068
No health risk factors	.004 (.003, .013)	.001 (.002, .003)	-.001 (.005, .003)	.000 (-.001, .001)
Counterfactual outcome	.081	.026	.022	.003
Observations	178,009	184,036	236,838	243,195
Health risk factor	-.003 (.015, .009)	-.003 (.008, .003)	-.003 (.009, .005)	.001 (-.001, .003)
Counterfactual outcome	.221	.058	.065	.009
Observations	175,741	229,484	152,852	202,407
	**I: Inpatient stay (ACSC)**	**J: Psychotropic drug use**	**K: Antibiotic use**	
Full sample	-.001 (-.002, .001)	.000 (-.008, .007)	.001 (-.003, .006)	
Counterfactual outcome	.007	.138	.078	
Observations	406,247	394,754	293,658	
No health risk factors	.000 (-.001, .000)	.001 (-.006, .007)	.001 (-.004, .007)	
Counterfactual outcome	.002	.093	.058	
Observations	168,877	227,214	181,936	
Health risk factor	-.001 (-.004, .002)	-.002 (-.013, .011)	.001 (-.006, .01)	
Counterfactual outcome	.012	.186	.099	
Observations	189,398	200,782	157,882	

Notes: ⁎p*<*0.1; ⁎⁎p*<*0.05; ⁎⁎⁎p*<*0.01. This table shows the conventional RD estimates for the three-months pre-period within the MSE-optimal window, produced separately for each outcome using triangular kernel. The robust bias corrected confidence intervals are shown in parentheses, which are based on the standard errors clustered at the month-year of birth level. Note that the robust confidence intervals are not necessarily centered around the conventional RD estimates. Individuals born in the second quarter of 1950 have been excluded from the sample due to the ambiguous treatment status. The results are presented for the full sample and the sub-samples based on the health risk group status. For each estimate, the counterfactual outcome and the number of observations are displayed. The counterfactual outcome indicates the conventional local polynomial left estimate, which refers to the estimated outcome for the control group at the threshold. The results were derived with Stata using the rdrobust package [42]

**Table 6:** Robustness check: Impact of the lockdown on healthcare use and mortality, using triangular kernel

	**A: In-person primary care visit**	**B: Remote primary care visit**	**C: Dental care visit**	**D: Physiotherapy visit**
Full sample	-.016** (-.032, -.003)	.002 (-.012, .012)	-.008 (-.013, .003)	-.003** (-.006, 0)
Counterfactual outcome	.298	.299	.152	.021
Observations	224,689	255,672	205,883	395,795
No health risk factors	-.012 (-.033, .008)	-.004 (-.024, .012)	-.013*** (-.018, -.003)	-.001 (-.004, .002)
Counterfactual outcome	.24	.231	.156	.016
Observations	117,819	132,888	114,436	204,769
Health risk factor	-.018*** (-.033, -.006)	.01 (-.003, .024)	-.004 (-.012, .01)	-.006*** (-.011, -.002)
Counterfactual outcome	.36	.369	.148	.027
Observations	124,122	140,320	114,675	178,727
	**E: Non-acute specialized care visit**	**F: Emergency department visit**	**G: Inpatient stay**	**H: Emergency department visit (ACSC)**
Full sample	-.01** (-.018, -.001)	-.001 (-.004, .003)	.000 (-.003, .004)	.000 (-.001, .001)
Counterfactual outcome	.14	.046	.042	.007
Observations	378,290	335,009	303,989	435,779
No health risk factors	-.006 (-.012, .003)	-.001 (-.005, .003)	.000 (-.005, .003)	.000 (-.001, .002)
Counterfactual outcome	.086	.032	.025	.003
Observations	188,212	203,598	205,812	184,317
Health risk factor	-.014*** (-.025, -.004)	-.001 (-.006, .005)	.000 (-.003, .006)	-.001 (-.003, .001)
Counterfactual outcome	.198	.061	.06	.011
Observations	199,157	159,412	148,535	179,445
	**I: Inpatient stay (ACSC)**	**J: Psychotropic drug use**	**K: Antibiotic use**	**L: Death during the lockdown**
Full sample	-.001 (-.002, .001)	-.002 (-.01, .006)	.001 (-.005, .007)	.000 (-.001, .001)
Counterfactual outcome	.007	.148	.08	.005
Observations	424,558	424,164	302,405	425,058
No health risk factors	-.001 (-.002, 0)	-.001 (-.008, .005)	-.001 (-.008, .004)	.001 (-.001, .002)
Counterfactual outcome	.003	.101	.061	.002
Observations	233,065	233,743	246,046	257,512
Health risk factor	-.001 (-.003, .002)	-.003 (-.014, .011)	.006** (0, .016)	-.001 (-.004, .001)
Counterfactual outcome	.011	.197	.099	.009
Observations	207,479	209,428	128,776	184,398

Notes: ⁎p*<*0.1; ⁎⁎p*<*0.05; ⁎⁎⁎p*<*0.01. This table shows the conventional RD estimates within the MSE-optimal window, produced separately for each outcome using triangular kernel. The robust bias corrected confidence intervals are shown in parentheses, which are based on the standard errors clustered at the month-year of birth level. Note that the robust confidence intervals are not necessarily centered around the conventional RD estimates. Individuals born in the second quarter of 1950 have been excluded from the sample due to the ambiguous treatment status. The results are presented for the full sample and the sub-samples based on the health risk group status. For each estimate, the counterfactual outcome and the number of observations are displayed. The counterfactual outcome indicates the conventional local polynomial left estimate, which refers to the estimated outcome for the control group at the threshold. The results were derived with Stata using the rdrobust package [42]

**Table 7:** Robustness check: Differences in health care use and mortality during the three-months post-period (Jul 2020–Sep 2020), using triangular kernel

	**A: In-person primary care visit**	**B: Remote primary care visit**	**C: Dental care visit**	**D: Physiotherapy visit**
Full sample	-.016* (-.042, 0)	-.007*** (-.016, -.004)	.006 (-.002, .012)	.002 (.000, .005)
Counterfactual outcome	.342	.266	.22	.027
Observations	182,907	160,486	410,447	344,969
No health risk factors	-.018* (-.043, 0)	-.007*** (-.018, -.003)	.004 (-.009, .014)	.002 (-.003, .006)
Counterfactual outcome	.287	.21	.223	.019
Observations	93,986	93,356	153,174	165,889
Health risk factor	-.013 (-.038, .004)	-.004 (-.015, .004)	.007 (-.004, .017)	.001 (-.005, .007)
Counterfactual outcome	.401	.325	.215	.036
Observations	108,157	118,225	199,964	133,860
	**E: Non-acute specialized care visit**	**F: Emergency department visit**	**G: Inpatient stay**	**H: Emergency department visit (ACSC)**
Full sample	-.002 (-.01, .005)	-.002 (-.007, .002)	.001 (-.002, .005)	.000 (-.002, .001)
Counterfactual outcome	.15	.048	.04	.006
Observations	397,918	447,509	272,684	387,115
No health risk factors	.001 (-.005, .006)	.001 (-.005, .006)	.001 (-.003, .004)	.000 (-.001, .002)
Counterfactual outcome	.094	.033	.024	.003
Observations	267,788	237,808	180,193	287,686
Health risk factor	-.004 (-.017, .01)	-.004 (-.01, .004)	.002 (-.002, .009)	-.001 (-.003, .002)
Counterfactual outcome	.21	.062	.054	.010
Observations	204,506	157,393	151,404	177,057
	**I: Inpatient stay (ACSC)**	**J: Psychotropic drug use**	**K: Antibiotic use**	**L: Death during the post-period**
Full sample	.000 (-.001, .002)	-.001 (-.008, .006)	.000 (-.005, .005)	.001 (.000, .002)
Counterfactual outcome	.006	.137	.066	.003
Observations	306,482	399,218	320,899	259,948
No health risk factors	.001** (0, .002)	.000 (-.006, .007)	.002 (.003, .008)	.000 (-.001, .002)
Counterfactual outcome	.002	.093	.048	.001
Observations	144,626	207,962	184,379	205,086
Health risk factor	-.001 (-.004, .002)	-.002 (-.015, .011)	-.003 (-.009, .004)	.001* (.000, .004)
Counterfactual outcome	.010	.183	.086	.004
Observations	164,343	205,843	161,402	124,890

Notes: ⁎p*<*0.1; ⁎⁎p*<*0.05; ⁎⁎⁎p*<*0.01. This table shows the conventional RD estimates for the three-months post-period within the MSE-optimal window, produced separately for each outcome using triangular kernel. The robust bias corrected confidence intervals are shown in parentheses, which are based on the standard errors clustered at the month-year of birth level. Note that the robust confidence intervals are not necessarily centered around the conventional RD estimates. Individuals born in the second quarter of 1950 have been excluded from the sample due to the ambiguous treatment status. The results are presented for the full sample and the sub-samples based on the health risk group status. For each estimate, the counterfactual outcome and the number of observations are displayed. The counterfactual outcome indicates the conventional local polynomial left estimate, which refers to the estimated outcome for the control group at the threshold. The results were derived with Stata using the rdrobust package [42]

**Table 8:** Robustness check: Placebo tests for outcomes during the lockdown, cutoffs at 65 and 75

	Cutoff = 65	Cutoff = 75	Cutoff = 65	Cutoff = 75
	**A: In-person primary care visit**		**B: Remote primary care visit**	
Full sample	.005 (-.005, .014)	-.039*** (-.076, -.014)	-.003 (-.013, .004)	-.008 (-.027, .008)
Counterfactual outcome	.258	.369	.246	.358
Observations	204,795	185,118	354,911	210,485
	**C: Dental care visit**		**D: Physiotherapy visit**	
Full sample	-.003 (-.009, .005)	.001 (-.003, .008)	.000 (-.002, .002)	-.001 (-.003, .002)
Counterfactual outcome	.183	.130	.019	.018
Observations	293,850	199,974	310,781	183,811
	**E: Non-acute specialized care visit**		**F: Emergency department visit**	
Full sample	-.001 (-.006, .006)	-.003 (-.009, .004)	-.001 (-.005, .002)	-.002* (-.006, .000)
Counterfactual outcome	.120	.159	.038	.057
Observations	228,912	263,197	389,363	353,539
	**G: Inpatient stay**		**H: Emergency department visit (ACSC)**	
Full sample	-.001 (-.004, .002)	-.003* (-.007, .000)	.000 (-.001, .001)	.000 (-.002, .001)
Counterfactual outcome	.033	.057	.004	.008
Observations	288,044	237,801	326,722	421,585
	**I: Inpatient stay (ACSC)**		**J: Psychotropic drug use**	
Full sample	.000 (-.002, .001)	.000 (-.002, .002)	.000 (-.008, .008)	-.001 (-.010, .006)
Counterfactual outcome	.004	.009	.141	.167
Observations	348,919	282,788	366,897	355,527
	**K: Antibiotic use**		**L: Death during the lockdown**	
Full sample	-.002 (-.006, .002)	.000 (-.006, .005)	.000 (-.001, .001)	-.001 (-.002, .001)
Counterfactual outcome	.079	.091	.003	.008
Observations	418,387	288,301	311,029	358,677

Notes: ⁎p*<*0.1; ⁎⁎p*<*0.05; ⁎⁎⁎p*<*0.01. This table shows the conventional RD estimates within the MSE-optimal window, produced separately for each outcome using uniform kernel. In this table, cutoffs are set to 65 and 75 to consider the plausibility of the actual cutoff at age 70. The robust bias corrected confidence intervals are shown in parentheses, which are based on the standard errors clustered at the month-year of birth level. Note that the robust confidence intervals are not necessarily centered around the conventional RD estimates. For each estimate, the counterfactual outcome and the number of observations are displayed. The counterfactual outcome indicates the conventional local polynomial left estimate, which refers to the estimated outcome for the control group at the threshold. The results were derived with Stata using the rdrobust package [42]

**Table 9:** Robustness checks: Impact of the lockdown on the number of healthcare visits and drug purchases (count data)

	**A: In-person primary care visit**	**B: Remote primary care visit**	**C: Dental care visit**	**D: Physiotherapy visit**
Full sample	-.036*** (-.065, -.013)	.009 (-.028, .040)	-.026*** (-.036, -.008)	-.006* (-.013, .001)
Counterfactual outcome	.583	.632	.236	.037
Observations	160,240	194,621	236,598	303,087
No health risk factors	-.021 (-.057, .008)	.021 (-.020, .056)	-.038*** (-.050, -.024)	-.005 (-.011, .002)
Counterfactual outcome	.416	.426	.245	.029
Observations	96,970	100,171	197,154	211,218
Health risk factor	-.022 (-.062, .032)	.004 (-.036, .047)	-.022* (-.039, .003)	-.011 (-.023, .004)
Counterfactual outcome	.758	.842	.232	.049
Observations	92,072	109,002	126,496	124,122
	**E: Non-acute specialized care visit**	**F: Emergency department visit**	**G: Inpatient stay**	**H: Emergency department visit (ACSC)**
Full sample	-.037*** (-.072, -.012)	-.003 (-.008, .003)	.007 (-.056, .089)	-.001 (-.002, .001)
Counterfactual outcome	.313	.060	.359	.008
Observations	401,477	318,390	279,930	333,759
No health risk factors	-.023** (-.049, -.002)	.001 (-.004, .006)	-.016 (-.078, .052)	.000 (-.001, .002)
Counterfactual outcome	.155	.038	.189	.004
Observations	137,229	171,218	157,054	156,332
Health risk factor	-.051** (-.109, -.008)	-.008* (-.017, .001)	.018 (-.109, .167)	-.001 (-.004, .002)
Counterfactual outcome	.476	.082	.548	.012
Observations	207,398	182,310	133,982	146,123
	**I: Inpatient stay (ACSC)**	**J: Psychotropic drug use**	**K: Antibiotic use**	
Full sample	-.012* (-.029, .001)	-.014 (-.061, .028)	-.006 (-.015, .003)	
Counterfactual outcome	.051	.465	.111	
Observations	365,112	310,482	280,857	
No health risk factors	.000 (-.012, .010)	.016 (-.021, .052)	.002 (-.007, .009)	
Counterfactual outcome	.014	.255	.075	
Observations	164,473	160,323	208,413	
Health risk factor	-.021* (-.059, .005)	-.022 (-.099, .067)	-.006 (-.021, .010)	
Counterfactual outcome	.088	.681	.144	
Observations	170,504	131,370	143,367	

Notes: ⁎p*<*0.1; ⁎⁎p*<*0.05; ⁎⁎⁎p*<*0.01. This table shows the conventional RD estimates within the MSE-optimal window, produced separately for each outcome using uniform kernel. Unlike in our main analyses, these estimates are based on count data, reflecting the effect of the lockdown on the number of visits and drug purchases. The robust bias corrected confidence intervals are shown in parentheses, which are based on the standard errors clustered at the month-year of birth level. Note that the robust confidence intervals are not necessarily centered around the conventional RD estimates. Individuals born in the second quarter of 1950 have been excluded from the sample due to the ambiguous treatment status. The results are presented for the full sample and the sub-samples based on the health risk group status. For each estimate, the counterfactual outcome and the number of observations are displayed. The counterfactual outcome indicates the conventional local polynomial left estimate, which refers to the estimated outcome for the control group at the threshold. The results were derived with Stata using the rdrobust package [42]

**Table 10:** Robustness checks: Multiple hypothesis testing

	RD: Conventional	RD: Robust	Romano-Wolf	Holm
Non-acute care				
A: In-person primary care visit	0.003	0.010	0.025	0.008
B: Remote primary care visit	0.990	0.970	0.969	0.969
C: Dental care visit	0.001	0.022	0.042	0.096
D: Physiotherapy visit	0.010	0.031	0.042	0.012
E: Non-acute specialized care visit	0.000	0.001	0.005	0.010
Acute care				
F: Emergency department visit	0.481	0.547	0.903	1.000
G: Inpatient stay	0.799	0.647	0.913	1.000
H: Emergency department visit (ACSC)	0.579	0.651	0.913	1.000
I: Inpatient stay (ACSC)	0.953	0.993	0.995	0.995
Medication use				
J: Psychotropic drug use	0.972	0.989	0.991	0.987
K: Antibiotic use	0.803	0.932	0.991	1.000
Mortality				
L: Death during the lockdown	0.791	0.922	0.895	0.895

Notes: ⁎p*<*0.1; ⁎⁎p*<*0.05; ⁎⁎⁎p*<*0.01. This table shows the results for multiple hypothesis testing. The first column reports the conventional (non-bias-corrected) p-values for the RD-estimates within the MSE-optimal window. The second column reports the robust bias-corrected p-values for the RD estimates within the MSE-optimal window, which are used for the inference throughout the paper. These RD estimations are performed using uniform kernel and clustering standard errors at the month-year of birth level. The third column reports the p-values based on the Romano-Wolf multiple hypothesis correction with 1000 bootstrap replications [43]. The fourth column additionally reports the p-values based on the Holm multiple hypothesis correction [65]. The multiple hypothesis testing is applied separately within each family (non-acute care, acute care, medication use, mortality), yielding family-wise error-rate–adjusted p-values that appropriately account for multiple testing while taking account for the logical grouping of outcomes. The results were derived with Stata using the rdrobust and rwolf2 packages [42, 66]

### Additional Tables

**Table 11:** Impact of the lockdown on dental care visits (public and private visits separately)

	A: Public dental care visit	B: Private dental care visit
Full sample	-.003 (-.007, .001)	-.01*** (-.016, -.003)
Counterfactual outcome	.071	.085
Observations	200,304	334,382
No health risk factors	-.007* (-.012, .001)	-.011*** (-.016, -.003)
Counterfactual outcome	.067	.096
Observations	138,556	154,933
Health risk factor	.002 (-.004, .009)	-.011** (-.02, -.002)
Counterfactual outcome	.074	.076
Observations	102,967	165,498

Notes: ⁎p*<*0.1; ⁎⁎p*<*0.05; ⁎⁎⁎p*<*0.01. This table shows the conventional RD estimates within the MSE-optimal window, produced separately for both outcomes using uniform kernel. The robust bias corrected confidence intervals are shown in parentheses, which are based on the standard errors clustered at the month-year of birth level. Note that the robust confidence intervals are not necessarily centered around the conventional RD estimates. Individuals born in the second quarter of 1950 have been excluded from the sample due to the ambiguous treatment status. The results are presented for the full sample and the sub-samples based on health the risk group status. For each estimate, the counterfactual outcome and the number of observations are displayed. The counterfactual outcome indicates the conventional local polynomial left estimate, which refers to the estimated outcome for the control group at the threshold. The results were derived with Stata using the rdrobust package [42]

**Table 12:** Impact of the lockdown on healthcare use (Sub-samples for Uusimaa region and rest of Finland)

	**A: In-person primary care visit**	**B: Remote primary care visit**	**C: Dental care visit**	**D: Physiotherapy visit**
Full sample	-.018** (-.033, -.004)	.000 (-.015, .014)	-.011** (-.017, -.001)	-.003** (-.006, 0)
Counterfactual outcome	.297	.298	.154	.021
Observations	170,561	177,219	200,304	298,594
Uusimaa region	-.022** (-.043, -.004)	-.002 (-.017, .017)	-.004 (-.015, .016)	-.001 (-.006, .005)
Counterfactual outcome	.238	.280	.156	.016
Observations	50,336	53,227	60,838	76,073
Rest of Finland	-.013 (-.031, .004)	.004 (-.014, .021)	-.014*** (-.019, -.005)	-.004*** (-.007, -.002)
Counterfactual outcome	.316	.303	.153	.023
Observations	126,490	138,449	164,062	242,618
	**E: Non-acute specialized care visit**	**F: Emergency department visit**	**G: Inpatient stay**	**H: Emergency department visit (ACSC)**
Full sample	-.012*** (-.019, -.005)	-.001 (-.005, .003)	.000 (-.003, .005)	.000 (-.001, .001)
Counterfactual outcome	.141	.046	.041	.007
Observations	359,445	293,788	231,892	314,397
Uusimaa region	.001 (-.009, .015)	-.01*** (-.017, -.004)	-.005 (-.011, .001)	-.002** (-.005, 0)
Counterfactual outcome	.108	.055	.038	.009
Observations	51,828	74,606	82,172	75,955
Rest of Finland	-.013*** (-.021, -.004)	.001 (-.003, .005)	.002 (-.002, .007)	.000 (-.001, .001)
Counterfactual outcome	.151	.043	.043	.007
Observations	240,287	212,068	185,162	233,322
	**I: Inpatient stay (ACSC)**	**J: Psychotropic drug use**	**K: Antibiotic use**	**L: Death during the lockdown**
Full sample	.000 (-.002, .002)	.000 (-.008, .008)	-.001 (-.006, .005)	.000 (-.001, .001)
Counterfactual outcome	.006	.147	.080	.005
Observations	309,671	353,227	298,594	362,915
Uusimaa region	-.001 (-.004, .001)	-.006 (-.022, .006)	.002 (-.008, .016)	.001 (-.001, .004)
Counterfactual outcome	.006	.141	.080	.006
Observations	87,572	83,354	63,818	87,358
Rest of Finland	.000 (-.002, .002)	.001 (-.006, .010)	-.001 (-.007, .006)	.000 (-.002, .001)
Counterfactual outcome	.007	.149	.081	.005
Observations	192,431	237,316	244,532	227,977

Notes: ⁎p*<*0.1; ⁎⁎p*<*0.05; ⁎⁎⁎p*<*0.01. This table shows the conventional RD estimates within the MSE-optimal window, produced separately for each outcome using uniform kernel. The robust bias corrected confidence intervals are shown in parentheses, which are based on the standard errors clustered at the month-year of birth level. Note that the robust confidence intervals are not necessarily centered around the conventional RD estimates. Individuals born in the second quarter of 1950 have been excluded from the sample due to the ambiguous treatment status. The results are presented for the full sample and the sub-samples based on whether the individuals reside in the Uusimaa region or elsewhere in Finland. For each estimate, the counterfactual outcome and the number of observations are displayed. The counterfactual outcome indicates the conventional local polynomial left estimate, which refers to the estimated outcome for the control group at the threshold. The results were derived with Stata using the rdrobust package [42]

## Footnotes

1 According to current conventions in the RD literature, our exact estimation samples vary by outcome, as each outcome is estimated within a unique mean squared error (MSE) optimal bandwidth (see Section“Identification strategy” for details).

2 Our main results are not sensitive to this coding choice, as the estimates based on count data are consistent with our main findings (see Table 9).

3 This indicates setting bwselect(msetwo) in the rdrobust command.
